# Targeting Triple‐Negative Breast Cancer: Resistance Mechanisms and Therapeutic Advancements

**DOI:** 10.1002/cam4.70803

**Published:** 2025-05-02

**Authors:** Rachana Raman, Shristi Debata, Thirupugal Govindarajan, Praveen Kumar

**Affiliations:** ^1^ Photoceutics and Regeneration Laboratory, Department of Biotechnology, Centre for Microfluidics, Biomarkers, Photoceutics and Sensors (μBioPS), Manipal Institute of Technology Manipal Academy of Higher Education Manipal Karnataka India; ^2^ Innotech Manipal, Manipal Institute of Technology Manipal Academy of Higher Education Manipal Karnataka India; ^3^ Department of Biotechnology, Manipal Institute of Technology Manipal Academy of Higher Education Manipal Karnataka India; ^4^ Department of Internal Medicine UT Southwestern Medical Center Dallas Texas USA

**Keywords:** biomarkers, cancer stem cells, phytochemicals, plasticity, single‐cell RNA sequencing, tumor microenvironment

## Abstract

**Background:**

Triple‐negative breast cancer (TNBC) is one of the most heterogeneous and menacing forms of breast cancer, with no sustainable cure available in the current treatment landscape. Its lack of targets makes it highly unresponsive to various treatment modalities, which is why chemotherapy continues to be the primary form of treatment, despite the high rates of patients developing chemoresistance. In recent years, however, there has been significant progress in identifying and understanding the role of several aspects that might contribute to genomic instability and other hallmarks of cancer, including cellular proteins, immune targets, and epigenetic mechanisms, which are desirable as they permit reversibility easier than the often‐adamant genetic changes.

**Methods:**

A literature review was conducted on the role of various TNBC associated biomarkers, their therapeutic applications, and their role in tumorigenesis and tumor maintenance, with a focus on linking both the driving biological mechanisms and emerging treatment options for TNBC.

**Conclusions:**

Shifting the focus of treatment to identify crucial tumor cell subpopulations and associated biomarkers, such as local immune cell populations and cancer stem cells, could potentially solve or simplify decades' worth of problems that are associated with TNBC, bolstering early detection and the evolution of precision medicine and treatment. The techniques that can be used here are epigenetic analysis and RNA sequencing. Biomarkers, such as PD‐L1, survivin, and ABC transporters, are implicated in several crucial processes that maintain tumors, such as cell proliferation, metastasis, immunosuppression, and stemness. Complex treatment options such as, immunotherapy, pathway inhibition, PARP inhibition, virotherapy, and RNA targeting have been considered for TNBC. Phytochemicals are also being considered as a treatment modality for TNBC, as a supplement to chemotherapy and radiation therapy, or as sole treatment.

## 1 Introduction

Triple‐negative breast cancer (TNbc) is an aggressive subtype of breast cancer (bc) with a high histological grade, poor prognosis, early relapse, and increased metastasis. TNBC constitutes ~20% of BC. TNBC is named so because it lacks the expression of three normally prevalent tumor receptors, namely the estrogen receptor (ER), progesterone receptor (PR), and human epidermal growth factor receptor 2 (HER2). There are six major subtypes of TNBC [1], basal‐like 1 (BL1), basal‐like 2 (BL2), immunomodulatory (IM), a mesenchymal (M), a mesenchymal‐stem‐like (MSL), and a luminal androgen receptor (LAR) subtype [2]. The BL1 and BL2 types have a higher expression of cell cycle and DNA damage response genes. The M and MSL types show high epithelial to mesenchymal transition (EMT) with the involvement of several growth factor pathways. The IM subtype is mostly involved in immune cell processes (NK cell pathway, BCR signaling pathway, etc.). The LAR subtype is involved in AR signaling and has androgen receptor‐driven tumors [1].

Tumor recurrence is mainly attributed to cancer stem cells (CSCs) because they show resistance to treatment and proliferate at a much slower rate, making them difficult targets for several chemotherapeutic drugs. These drugs, which mainly target rapidly proliferating cells in the region, often miss out on CSCs, leading to the development of chemoresistance [3, 4]. Some tumors also show multi‐drug resistance due to transporter molecules, leading to improper tumor clearance and left‐over tumor initiating cells. Mutations of tumor suppressor genes like TP53 or BRCA and upregulation of oncogenes like MYC have also been associated with different subtypes of TNBC. The biomarkers, several of which are overexpressed or downregulated specifically in the case of TNBC, can be potential target sites to create novel treatments alongside chemotherapy. The development of certain new technologies like single‐cell RNA sequencing and analysis of the epigenetic environment of cancer cells is gaining momentum to improve the avenues for TNBC treatment.

Current standard‐of‐care (SOC) chemotherapy includes anthracyclines, alkylating agents, microtubule inhibitors with or without Taxane, anti‐metabolites, platinum agents, and DNA crosslinkers [5]. Another treatment option is neoadjuvant chemotherapy, followed by surgery [6]. But in the case of large tumors or relapsed/refractory TNBCs, there are no established SOC treatment regimens. Treatment responses tend to wear off soon and are followed by relapse and visceral and brain metastases. In early‐stage TNBC, if the tumor is small enough, surgery is the primary treatment. For larger tumors and cancers of lymph nodes, surgery is often followed by radiation. But in the case of higher grade TNBCs, chemotherapy is the standard treatment course. Less than one‐third of TNBCs are sensitive to SOC chemotherapy or hormonal therapy. This demands the need for new and innovative therapeutic strategies. In addition to the above, most drugs and therapies target ER, PR, and HER2, thus making it especially difficult to treat tumors that are not responsive to the usual hormones given in the case of non‐TNBCs. Therefore, basic anti‐HER2 therapy cannot be used [7] and targeted therapy is the need of the hour to treat and prevent recurrence and metastasis of TNBC tumors. For example, the presence of tumor infiltrating lymphocytes in the microenvironment and the high expression of programmed cell death‐ligand (PD‐L1) in TNBC makes immunotherapy another viable treatment option. While several factors, like the presence of CSCs and genetic mutations of tumor suppressor genes and oncogenes, are well‐known defining factors in TNBC, recent studies have investigated and propelled the need to identify the epigenetic mechanisms that drive tumor progression. Further, understanding the tumor microenvironment (TME) can be better achieved by studying the epigenetic landscape.

## 2 Therapeutic Resistance in TNBC

### 2.1 Development of Therapeutic Resistance due to CSCs

Normally, tissue resident stem cells help in adult tissue repair and maintenance. However, in some cancers, these stem cells dedicate themselves to the prolonged maintenance, recurrence, and metastasis of tumor cells even after their removal, and are called CSCs. CSCs have a strong self‐renewing potential, which contributes to tumorigenesis. When CSCs are transplanted into severe combined immuno‐deficient mice, they retain the ability to form tumors, unlike non‐CSCs. In 2003, Al Hajj et al. [8] described the markers specific to tumor initiating cells in BC. Not all cells have the potential to initiate tumors. They identified a subpopulation of cells with cell surface markers that were tumorigenic, which helps us to distinguish and target those cells effectively. These cells bore the CD44+/CD24− phenotype and had an innate ability to form heterogeneous tumors, much like the original tumor.

In 2006, Ginestier et al. [9] identified that tumor human mammary stem cells have increased aldehyde dehydrogenase (ALDH) activity. High ALDH1 activity is associated with the identification of tumorigenic cell fractions with high self‐renewing potential and similar heterogeneity as the parent tumor. In TNBC, the stem cells are usually of CD44+/CD24− phenotype with high ALDH1 activity, unlike non‐TNBC cells. In 2014, Liu et al. [10] identified that the CD44+/CD24− markers represent the mesenchymal‐like stem cells which are usually located at the periphery of the tumor, whereas the high ALDH activity represents the epithelial‐like stem cells that are localized more centrally. The plasticity of the stem cells, depending on the TME, allows for transition between the epithelial and mesenchymal states and vice versa, which helps in tissue invasion and metastasis. They established that the mesenchymal‐like stem cells are more quiescent and have a higher invasive capacity, whereas the epithelial‐like cells are mostly proliferative in nature.

CSCs are also one of the primary reasons for a tumor developing resistance to chemotherapy, which can be attributed to plasticity and its related processes. They are drivers of EMT, tumor recurrence, and metastasis and are an emerging target for therapy, with EMT‐related genes such as doublecortin‐like kinase 1 (DCLK1) or epigenetic targets emerging to the fore for therapeutic applications [11]. A few genes were found to be upregulated, thereby maintaining TNBC stemness. For example, BIRC5, TPX2, CDC25A, KIF2C, and RAD54L are a few key genes relevant to maintaining stemness [12]. In another clinical study, TPX2 was found to be overexpressed in nearly all the patients and indicated worse outcomes in terms of OS and tumor aggressiveness [13]. TPX2 inhibition, in turn, negatively regulates the PI3K/Akt pathway and enhances cell death via apoptosis, thereby reducing cancer cell proliferation [14]. CDC25A is another cell cycle regulator that has been identified as an oncogene promoting tumor proliferation, invasion, and association with recurrence in BCs [12]. A bioinformatics analysis identified kinesin superfamily (KIF) genes enriched during DNA replication and cell cycle processes as a proliferation marker for TNBC [12]. Although such bioinformatics analyses have provided us with evidence to regulate such genes to control cancer stemness, extensive research is needed to confirm the analyses.

In TNBC, tumors are enriched with CD44+/CD24− stem cells, which are more aggressive and tumorigenic than non‐CD44+/CD24− cell types. The dormant nature of CSCs has shielded them against all cytotoxic drugs currently in use. Certain molecules or enzymes that play a crucial role in processes that aid stem cell proliferation and maintenance, like EMT can be targeted by inhibitor molecules. For example, GSK3β inhibition has been shown to reduce the rate of EMT and suppress CSCs by downregulating the enzyme [15]. An example of a protein target could be the TRIM28 protein, whose knockdown has shown a significant decrease in certain pluripotent and mesenchymal markers that drive CSC population expansion and reduce their number while also inducing tumor shrinkage [16]. Another treatment option targeting CSC markers or cell surface antigens is JQ1, a bromo‐domain and extra‐terminal domain family (BET) protein inhibitor, which targets several genes regulating stemness and suppresses the expression of CD44/CD24 ratios and ALDH activity. It also suppresses the effects of several transcription factors RUNX2, MYC, etc., which are responsible for the maintenance of CSCs [17]. Hyaluronic acid, a natural ligand for the CD44 receptor, is used to coat nanoparticles to deliver chemotherapy drugs for better efficacy and clearance of TNBC [18]. Several signaling pathways are also involved in the maintenance of CSCs, which can serve as potential targets for treatment. BIRC5 encodes a protein called survivin, which is responsible for cell cycle regulation, mitosis, and apoptosis. The overexpression of this protein in tumors is associated with reduced cell death and is found to indicate a poor prognosis in several cancers, making it a great prognostic factor and therapeutic target [19]. While a large array of biomarkers is associated with CSCs, a few challenges remain persistent when it comes to specifically targeting CSCs. These challenges include increased vasculogenesis, which can lead to difficulties in drug penetration and delivery, and the high degree of heterogeneity in a single population, rendering systems that can target only a limited number of markers ineffective [20]. These problems may be overcome by employing highly precise and compact delivery systems, such as viruses or nanocarrier systems, the former of which has been discussed in later sections. Thus, CSCs are the primary cells responsible for the resistance to treatment, and the associated mechanisms and implications for therapy are highlighted below.

### 2.2 Cellular Plasticity and Its Role in Chemoresistance

Cellular plasticity is an important event that allows cells to adapt and switch into different phenotypes according to the microenvironment; for example, EMT or switching into stem cell‐like properties [21]. Sometimes, they also transform into endothelial cells to form blood vessels that accommodate tumor cells [21]. The cytokines present in the TME often promote the conversion of cells into CSCs. Oncostatin‐M, a TME cytokine, leads to the de‐differentiation of tumor cells into CSCs and can be suppressed by enhancing the Interferon‐β (IFN‐β) signaling process, which is often regulated by the resident immune cells. De‐differentiation is also seen in chemotherapy. Although IFN‐β has shown good results in other forms of cancers, its dosage levels to reduce toxic effects are yet to be established in TNBC [22]. Some factors, like hypoxia, induce cellular plasticity with chemoresistance and aggressive malignant phenotypes [23]. In a 2019 study, Samanta et al. [24] found that treatment of TNBC with paclitaxel or gemcitabine induces higher hypoxia‐inducing factors (HIF) activity, which promotes transcriptional activity in TNBC and thereby leads to its enrichment. Hypoxia leads to treatment resistance as it creates an acidic microenvironment that makes drugs less effective and reduces their uptake [25]. HIF‐1 regulates the expression of certain genes, which leads to enhanced tumor aggressiveness and also contributes to the downregulation of adhesion molecules, leading to metastasis [23]. HIF‐1 induces the upregulation of carbonic anhydrase (CA) IX, which enhances hypoxia and leads to chemoresistance and the development of more aggressive tumor phenotypes [26]. HIF inhibitors have been used in treating renal cancer and could be used to treat TNBC. Inhibition of these factors will help sensitize the tumors to chemotherapy.

### 2.3 Role of Survivin in TNBC

Survivin is an important protein implicated in the prognosis and treatment of several cancers, and it works by interfering with Caspase 3 activity during the G2/M phase of mitosis [27]. It belongs to the Inhibitors of apoptosis proteins (IAPs) family, which is a group of proteins capable of suppressing NAIP, etc. Survivin inhibits the apoptosis of cancer cells and is present in elevated levels near the tumor area, in serum, and in urine [28]. Survivin has been established in several cancers as a diagnostic marker; for example, colon, prostate, and hematological cancers, and it has been implicated in aiding tumor survival of CSCs under unfavorable conditions as well [19]. BIRC5 encodes survivin, which is responsible for cell cycle regulation, mitosis, and apoptosis. The overexpression of this protein in tumors is associated with reduced cell death and is found to indicate a bad prognosis in several cancers, such as glioblastoma (GBM), where overexpression of BIRC5 has been associated with a poor prognosis, as the gene itself has been implicated in promoting the formation of CSCs and maintaining stemness [29], thus making survivin a great prognostic factor and therapeutic target.

In TNBC, elevated survivin levels are associated with a poor prognosis. In a 2019 study conducted with 150 specimens, cancerous tissues were found to have 61.4% more survivin content compared to normal tissues. Rates of overall survival (OS) and disease‐free survival were also found to decrease with increasing levels of survivin and decreased levels of ZIC‐1 [30]. The overexpression of TP‐dependent dynamin‐related proteins such as Drp‐1 silences Notch‐1 proteins that can induce apoptosis and increase the rate of mitochondrial division. This, in turn, upregulates survivin levels in tumor tissue and helps in cell proliferation and tumor progression and is associated with a poor prognosis [31] TGF‐β1 belongs to the family of immunosuppressive cytokines and is secreted by tumor‐associated macrophages (TAMs) and is also implicated in aiding endothelial to mesenchymal transition (EMT) [32]. A combined assessment of TGF‐β1 and survivin levels in TNBC patients is elevated compared to normal levels and is associated with poor OS and PFS [32].

Survivin is an emerging target for the treatment options discussed above. The role of survivin as a biomarker marks it as an important target for TNBC treatment. It contributes to chemoresistance and is known to be selectively expressed and regulated by cancerous cells [19]. Selinexor was found to inhibit survivin expression by two methods: initiating nuclear accumulation of survivin, which is eventually digested by intracellular proteases, and by inhibiting survivin gene transcription [33]. However, the efficacy of Selinexor as a monotherapy is limited. A Phase II clinical trial showed that the median OS was 5.98 months [34]. The usage of miR‐34a is shown to downregulate survivin and improve selinexor efficacy [33]. Additional methods of survivin targeting include knockdown of export proteins such as XPO1 [35] and MX106/MX107 in conjunction with chemotherapy [36].

### 2.4 Role of ABC Transporters in Chemoresistance

A number of reasons can be cited for the development of chemoresistance in TNBC, but transporter‐mediated drug efflux remains a consistent one. ATP‐binding cassette (ABC) transporters use ATP to pump out several compounds across the cell membrane and contribute to multidrug resistance (MDR) [25]. In this process, many anticancer drugs also move out, leading to chemoresistance. Three of the main ABC transporters that are highly expressed in TNBC are ABCC1/MRP1 (multidrug‐resistant protein 1), ABCC11/MRCP8 (MDR protein 8) and ABCG2/BRCP (BC resistance protein) [37, 38]. It has been observed that patients with high levels of ABCC1 show decreased pathological complete response (pCR). ABCC1 protein expression increases following chemotherapy, leading to residual disease and low pCR in TNBC [39]. ABCG2 also contributes to the chemoresistance of stem cells in TNBC. It has been observed that the knockdown of the growth hormone receptor leads to the downregulation of ABCG2, sensitizing the tumors to chemotherapy [40]. ABCC1 resists many chemotherapy drugs like taxanes, anthracyclines, methotrexates, etc., whereas ABCG1 effluxes drugs 5‐fluorouracil, methotrexate, doxorubicin, etc. All three ABC transporters share many common substrate specificities and therefore confer resistance to several chemotherapy drugs in TNBC.

In order to sensitize tumors to chemotherapy, we need to target ABC transporters, which can be done in two ways—either inhibiting the transporters or inhibiting their expression. PZ‐39 is an inhibitor of ABCG2 that degrades as well as inhibits the transporter [41]. Side population (SP) cells also express ABCG2/BRCP1 [42] which confers resistance to the drug mitoxantrone. ABCG2 targeting could be a potential strategy to treat the tumors. However, there are no approved ABCG2 inhibitor drugs so far. Nanoparticle drug delivery systems have also shown promising results in inhibiting ABCC1 and ABCG2. Paclitaxel, an extensively used chemotherapy drug, has been used in an indomethacin‐based micelle to treat MDR [43]. RNA interference (RNAi) is another technique that needs to be explored to target the expression of these transporters. Small interfering RNA (siRNA) and micro RNA (miRNA) can be used to target and downregulate the expression of these genes, which in turn will effectively reduce MDR in tumors. There are also several cellular signaling pathways that interact and contribute to cell plasticity, some of which are described below. There are also several cellular signaling pathways that interact and contribute to chemoresistance, some of which are described in Section 4.

## 3 Role of Epigenetics in TNBC

### 3.1 Role of Epigenetic Mechanisms in Inhibiting Tumorigenesis

The prospect of reversing the mutations caused by epigenetic modifications seems like a promising approach in managing several cancers, TNBC being no exception. Targeting epigenetic modifications, genes, and signaling pathways has become a matter of critical investigation and research over the past years. A lot of research has been published in areas of DNA methylation, histone modifications, and non‐coding RNAs, which opens avenues for targeted therapy. The integrated omics approach has emerged as a powerful technique to study thousands of gene sequences, in addition to the purpose of comparing and identifying interactions between large‐scale genetic and epigenetic data unique to TNBC [44]. Databases like *The Cancer Genome Atlas* (TCGA) are extremely helpful in analyzing data pertaining to TNBC tumors and have helped reveal the effects of epigenetic variations on signaling pathways and networks harboring mutations. Aberrant DNA methylation patterns in genes BRCA, P53, ATM, FANC, etc., which are important DNA damage repair genes, have been found to predispose a person to TNBC [45]. Epigenetic alterations can also drive tumor metastasis by the process of EMT. Long non‐coding RNAs (lncRNAs), miRNAs, methylation, and other histone modifications influence transcription factors that affect pathway signaling and lead to metastasis. Some lncRNAs like GAS5, ARNILA, UCAT, and NAMPT‐AS are directly found to be associated with EMT pathways, which can work toward sustaining and transcribing pro‐tumor genes [46, 47]. Similarly, miRNAs like miR‐26a and miR‐10b, among several others, have prognostic values and can be treated as potential biomarkers contributing to anti‐ or pro‐tumoral activity [46].

Due to developments around the interactions between epigenetics and genetics, some success has also been obtained in trying to control metastatic drivers in TNBC. Epithelial reprogramming has been achieved by using DNA methyltransferase (DNMT) and histone deacetylase (HDAC) inhibitors, like SGI‐110 and MS275 respectively, which work to reduce cellular proliferation and EMT by suppressing WNT signaling and mutant p53 expression [48]. GSK3β, which is another important kinase and a part of the WNT/NFkB signaling, is associated with poor prognosis in TNBC patients. GSK3β inhibitors, like BIO, have shown results wherein they selectively eliminate cells with mesenchymal properties and reduced stemness [15]. Integrated omics analysis of the signaling pathways has also led to an understanding the role of epoxyeicosatrienoic acids (EETs) and CYP‐epoxygenase, whose overexpression drives tumor metastasis [49]. Further research integrating both genetic and epigenetic aspects could certainly be the next big step in TNBC prognosis and treatment.

### 3.2 Analysis of Epigenetic Profiles in TNBC

The role of epigenetics in the regulation and prevention of several forms of cancers has been extensively studied in order to control and prevent certain types of cancers. Abnormal gene regulation due to epigenetic mechanisms can not only lead to tumor initiation but can also lead to CSC proliferation.

BMI1 and EZH2 are also two proteins whose epigenetic regulation plays an important role in cancer progression. An upregulated BMI1 leads to all tumor‐encouraging processes. The methylation patterns in the promoter region of PTEN may lead to the loss of tumor‐suppressive functions and encourage susceptibility to tumor formation [50]. Inhibitors of HDAC, DNMT, lysine‐specific deacetylase‐1 (LSD1), and BET proteins are being explored to reverse epigenetic modifications in cancer [17]. Several mechanisms, including chromatin remodeling and methylation patterns in promoter regions, may switch off certain tumor suppressor genes, leading to cancer [51]. Epigenetic markers like H3k9ac (a histone modification) associated with proliferation, migration, and other cell signaling processes have been found to be downregulated in TNBC [52]. Further, the hypomethylation in the promoter of the ST8SIA1 gene is responsible for the upregulation of the GD3S, which promotes proliferation and colony formation in TNBC [53]. Epigenetic analysis also plays a role in understanding treatment responses, as in cases where the methylome analysis brought out methylation signature patterns to predict pCR in patients. FERD3L and TRIP10 are two genes whose methylation patterns have been studied to predict the response to neoadjuvant chemotherapy. An inverse correlation was observed between methylation and gene expression, with an algorithm created to predict the response to neoadjuvant chemotherapy [54]. This type of algorithm can be very helpful to choose suitable therapies required across various patient populations. Epigenetic priming has also shown positive results where histone deacetylase inhibitors have been effective when used in combination with immunotherapy to enhance the effect of ICIs and decrease the growth of tumor cells [55]. Epigenetic reprogramming can also be carried out by modifying histone methylations, like in LSD1, which leads to an increase in chemokines attracting T cells and enhancing response [56]. Even in chemotherapy, drug resistance to Doxorubicin can be altered by HDAC inhibitors, where HDAC dysregulation can lead to tumorigenic conditions [57]. Certain epigenetic mechanisms regulate EMT processes. DNA methyltransferase and HDAC inhibitors, SGI and MS‐275, synergistically act to reprogram the epigenetic environment and suppress mutations in various pathways and genes to inhibit apoptosis and cellular proliferation, reiterating the role of epigenetics as a therapy option for TNBC. Hence, the role of ScRNA‐Seq and epigenetic modifications plays a pivotal role in developing future therapeutic targets and digging lesser known facts about TNBC.

## 4 Treatment Modalities for TNBC

### 4.1 Existing Treatment Options

Treatment options for TNBC have evolved considerably over the last few decades, but areas of concern remain in the choice of treatment regimens specific to each patient [5]. The standard regimen, consisting of chemotherapy (neoadjuvant or adjuvant) and surgery, considers several factors to determine a protocol suited to the individual cases, such as risk of recurrence, extent of tumor growth, subtype of cancer, age at the time of diagnosis, genetic history, and other suspected risk factors [5]. Genetic risk factors have been hypothesized to play an extremely important role in the choice of particular drugs. However, the TME of TNBC is highly diverse, and establishing a universal standard thus becomes implausible [58]. Surgery involves complete or partial resection of the breast tissue, with the primary aim being to preserve the original tissue [59]. Complete removal, that is, mastectomy, is a last resort in advanced cases. Surgical resection in TNBC can be tricky, owing to the highly metastatic and heterogeneous nature of the tumors [60]. Neoadjuvant treatments, administered via chemotherapy or immunotherapy, have been shown to improve surgical outcomes, and such a treatment regimen must be accompanied by adjuvant therapy [61].

Commonly used chemotherapeutic agents include anthracyclines, taxanes, paclitaxel, platinum, and fluorouracil‐based agents [62]. Anthracycline‐based chemotherapy (ATC) has been proven to be more effective for patients with Bcl2‐ tumors, but concerns about cardiomyopathy remain [63, 64]. Pertinent issues regarding chemotherapeutic drugs include their long‐term effects on patient quality of life, risk of relapse, and the dosage frequency. Standard dose densities have been shown to be less effective when compared to more dose‐dense regimens [65]. The substitution of chemotherapeutic drugs, such as taxanes, with platinum has been hailed as a potential solution to the development of toxic side effects associated with taxane‐based regimens [66, 67]. However, results regarding this modification remain largely inconclusive. For example, a Phase 3 trial involving postoperative patients revealed that the survival effects of using platinum‐based therapies were not significant, and the common claims pertaining to toxicity were dubious, as severe toxicity‐related effects were more common in patients treated with platinum alone [68].

Challenges associated with SOC treatments include the development of chemoresistance to the aforementioned treatments. Several molecular components, such as ABC transporters, confer resistance to chemotherapy [37]. The interference of several cellular pathways also confers chemoresistance and thus makes them viable targets for treating TNBC, as discussed in later sections. Other negative patient outcomes depend on the conditions of the patient prior to administration of chemotherapy. This includes amenorrhea in pregnancy‐associated BC, placental metastasis in pregnant patients, administration of palliative care leading to lower chances of survival in patients of advanced age, cardiomyopathy, impaired neurological function, higher incidence of leukemia, impaired glucose sensitivity that may progress to diabetes, and higher incidences of depression [69, 70, 71, 72, 73].

### 4.2 Advancements in Therapeutics

Therapeutic regimens have undergone several changes in the last few decades, with both improved regimens and novel therapeutics emerging as more suitable options to effectively treat TNBC and maintain an adequate patient quality of life post‐treatment.

#### 4.2.1 Combination Therapies

Treatment with singular therapeutic agents, also termed monotherapy, such as cyclophosphamide or melphalan, while effective, has several fatal or damaging side effects. For example, in a study of 90 patients diagnosed with cancer during a 10‐year period, it was found that melphalan or cyclophosphamide therapy, whether used alone or with adjuvants, was found to significantly increase the risk of leukemia after treatment, with elevated risks associated with treatment periods longer than 12 months or with melphalan treatment alone [74]. Combination therapies can counteract or subdue such toxic side effects, either by direct regulatory interactions with chemotherapeutic drug molecules or through lowered dosages achieved through the usage of less toxic therapeutics or more efficient delivery platforms [75, 76]. Novel combination therapies incorporate a few significant classes of drugs, including immunotherapeutic drugs, phytochemicals, pathway inhibitors, and nanomedicines.

##### 4.2.1.1 Chemotherapeutic Regimens

The administration of combination therapies in a neoadjuvant setting has risen in the last few years. An example of such regimens is the ones built around taxane as the primary drug. Taxanes, when used in combination with platinum, bevacizumab, and tabines, show an overall improved survival rate, reduced incidence of adverse side effects, such as anemia, alopecia, diarrhea, and vomiting, and reduce the risk of progressive disease [77]. Cyclophosphamide poses risks associated with fertility and cardiotoxicity but continues to remain a major drug for treating TNBC [78]. However, combination therapies, such as a regimen administering cyclophosphamide, carboplatin, and thiotepa, were found to be effective in treating TNBC through reduced chemoresistance owing to the presence of thiotepa, reduced toxicity due to lowered doses of cyclophosphamide, and an overall increase in progression‐free survival in patients aged 45 or younger [79]. Newer drugs, such as deruxtecan, are used in combination with datoputamab deruxtecan (dato‐DXd) show great promise in improving patient outcomes, especially with regard to treating unresectable tumors and combating metastasis [80]. Some emerging treatment modalities have been summarized in Table 1.

**TABLE 1:** Overview of clinical studies.

Clinical trial identifier	Regimen	Setting	Patient type	Study size	Completion status
NCT06393374	Sacituzumab tirumotecan and pembrolizumab	Adjuvant, interventional	Post‐operative patients who received neoadjuvant treatments	1530	Ongoing (start: 2024)
NCT02574455	Sacituzumab govitecan, eribulin, capecitabine, gemcitabine and vinorelbine	Adjuvant	Patients who have received at least two rounds of chemotherapy, with or without metastasis	529	Completed (duration: 2017–2020)
NCT04927884	N‐803, PD‐L1 t‐haNK, sacituzumab govitecan‐hziy and cyclophosphamide	Adjuvant, interventional	Patients without brain metastasis	3	Completed (2021–2022)
NCT06103864	Datopotamab deruxtecan (DATO‐DXd) with or without durvalumab	Neoadjuvant	PD‐L1 positive locally recurrent inoperable or metastatic TNBC	625	Ongoing (start: 2023)
NCT05374512	Dato‐DXd	Neoadjuvant	First‐line locally recurrent inoperable or metastatic TNBC	637	Ongoing (start: 2022)
NCT06112379	Dato‐DXd and durvalumab	Neoadjuvant, interventional	Patients with previously untreated TNBC or hormone receptor‐low/HER2‐negative breast cancer	1728	Ongoing (start: 2023)
NCT05629585	DATO‐DXd with or without durvalumab	Adjuvant, interventional	Post‐operative patients with metastasis in the lymph nodes after neoadjuvant chemotherapy	1075	Ongoing (start: 2022)
NCT04177108	Ipatasertib, atezolizumab, and paclitaxel	Neoadjuvant, interventional	Patients with previously untreated, unresected, locally advanced, metastatic TNBC	242	Completed (duration: 2019–2023)
NCT04251533	Alpelisib and nab‐paclitaxel	Neoadjuvant, interventional	Patients who have a PIK3CA mutation or PTEN loss	137	Ongoing (start: 2020)
NCT02819518	Pembrolizumab with nab‐paclitaxel, gemcitabine–carboplatin, or paclitaxel.	Adjuvant, interventional	Patients with locally recurrent or metastatic TNBC (inoperable), exhibiting high levels of PD‐L1	882	Completed (duration: 2016–2023)
NCT02574455	Sacituzumab govitecan	Adjuvant, interventional	Patients with relapsed or refractory TNBC	529	Completed (duration: 2017–2020)
NCT03036488	Pembrolizumab	Neoadjuvant and adjuvant treatment	Patients with untreated Stage II/III TNBC	1174	Ongoing (duration: February 17, 2025)

*Note:* Studies and trials from the past 5 years were considered for both subsections of the table. This includes those that were completed from 2019 onward and those that began from 2019 and after. Stage 3 clinical trials were considered.

Abbreviations: DATO‐DXd, datopotamab deruxtecan; nab‐paclitaxel, nanoparticle albumin‐bound paclitaxel; PIK3CA, phosphatidylinositol‐4,5‐bisphosphate 3‐kinase catalytic subunit alpha; PTEN, phosphate and tensin homolog; TNBC, triple‐negative breast cancer.

##### 4.2.1.2 Nanomedicines

In the cancer treatment landscape, nanomedicines have garnered significant interest as efficient delivery modules for various anti‐cancer drugs [81]. Several nanomedicines have been approved for TNBC treatment in the last decade, including the FDA‐approved abraxane (also referred to as nab‐paclitaxel) and doxil and the EMA‐approved myocet [82]. Nanocarriers serve to enhance the cell‐targeting, cell‐penetrating ability of therapeutics, in addition to improving their stability. For example, epigenetic drugs often face several issues when administered as monotherapies, including elevated rates of off‐targets, rapid clearance, and poor water solubility [83, 84]. Nanomicelle platforms can deliver drugs such as SAHA, which can overcome chemoresistance to tamoxifen in TNBC [83]. Nanomaterial‐based platforms help in improving cell‐targeting and cell‐penetrating abilities of drugs. This, in turn, reduces non‐specific cellular toxicity. Polymeric nanomaterials can be used in conjunction with doxorubicin and carboplatin to enhance drug clearance and eliminate off‐targets; however, limited clinical evidence in the area does not support any concrete conclusions, but studies such as the GeparSixto point toward areas for integrating nanomedicine in TNBC therapy [85, 86]. Several other chemotherapeutic drugs, such as docetaxel and paclitaxel, have also been combined with nanocarriers for enhanced therapeutic efficiency [87]. Abraxane has shown significantly better results in several clinical trials, with improved tolerance, survival rates, and reduced occurrence of side effects as compared to unbound paclitaxel, whether it is administered as a monotherapy or in conjunction with drugs such as carboplatin, gemcitabine, or bevacizumab [88].

The emergence of several novel therapeutic regimens has highlighted an underlying issue that must be resolved to obtain results of clinical relevance, that is, the identification of viable therapeutic targets in the solid tumor mass, TME, ECM, and surrounding tissue. The identification of suitable targets will serve to eliminate a significant amount of post‐operative and post‐treatment risks.

##### 4.2.1.3 Immunotherapy

Immunotherapy treatment modalities present a desirable solution to the issues currently prevalent in SOC treatment. Immunotherapy is most commonly implemented along with taxane or ATC, with the most prominent choices being atezolizumab and pembrolizumab, of which the latter currently has FDA approval for TNBC treatment [61, 89]. However, it is important to note that the implementation of immunotherapy in both adjuvant and neoadjuvant settings is still in its nascent stages, owing to several issues, including the lack of concrete evidence of its efficacy, doubts regarding long‐term effects on patient quality of life, costs, large differences in pCR among varied populations, and several incidences of immune‐related adverse events [90, 91, 92]. These events include the development of thyroid‐related disorders, for example, hyperthyroidism, adrenalitis, gastrointestinal conditions, among others [93, 94]. A more detailed discussion on the various targets available for immunotherapy, immunotherapeutic drugs, and relevant clinical trials is discussed in later sections.

## 5 Therapeutic Targets for TNBC

### 5.1 Cellular Signaling Pathways as Therapeutic Targets

Inhibition of the signaling pathways has shown promising results in terms of lowering tumor progression rates and improving chemosensitivity. Targeting the TGF‐β pathway is a strategy where small molecules are usually targeted at the TGF‐β receptor. The Phase 1 clinical trials of a growth factor inhibitor, galunisertib, along with paclitaxel, are being evaluated (NCT02672475). Monoclonal antibodies that target the pathway by being a ligand for the TGF‐β receptor can also be a potential therapeutic option that should be explored in the case of TNBC, as it has shown promising results for other types of cancers [95]. Another important method could be targeting anti‐sense TGF‐β oligonucleotide molecules. These single‐stranded molecules form complementary RNA transcripts that prevent the formation of certain TGF‐β ligands that inhibit the pathway [95]. Targeting the kinase activity of the TGF‐β receptors is another approach in which further signaling can be inhibited.

The hedgehog pathway is also an important pathway that is involved in the renewal of stem cells and tissue regeneration. In TNBC, it has a direct role in regulating the stem cell factors and promoting tumor growth, invasion, and metastasis. It also plays a role in drug resistance [96]. Smoothened (SMO) inhibitors are the main therapeutic targets in hedgehog signaling. Vismodegib and sonidegib are approved SMO inhibitor drugs that are used to treat basal cell carcinoma. The clinical trials of several similar drugs are ongoing to treat TNBC (NCT02694224). Sonidegib is an orally bioavailable drug to treat TNBC. Sonidegib, along with docetaxel, has shown positive results in clinical trials to treat advanced TNBC tumors [97]. Monoclonal antibodies to prevent the binding of sonic hedgehog (SHH) ligand to the PTCH receptor is also another therapy option that needs to be explored more. Certain chemotherapy drugs like doxorubicin induce notch signaling mediated MDR. The inhibition of this using γ secretase inhibitor, DAPT, is known to sensitize tumors to chemotherapy [98]. Another clinical trial has shown the efficacy of anti‐notch antibody tarextumab to inhibit the signaling in advanced tumors [99].

The Wnt/β‐catenin pathway is another pathway that may be overexpressed in TNBC. Benzimidazole compounds like SRI33576 and SRI35889 have been found to inhibit β‐catenin pathway with great specificity by downregulating LRP6, cyclin D1, and active β‐catenin in the nucleus [100]. Salinomycin, an antibiotic, has been reported to reduce the expression of the WNT/β‐catenin pathway, which reduced breast CSC proliferation and reduced mammosphere formation [101]. A recombinant peptide rhFzd7 inhibits the fzd7 receptor and promotes antitumor and anti‐angiogenic activity with great efficacy when combined with chemotherapy in TNBC [102]. Chlofazimine is another targeted therapy drug with low toxicity that has shown positive results in the suppression of TNBC cells inducing apoptosis and cell cycle arrest [103].

Inhibiting the NFkB pathway is also under process as many inhibitors have been identified. A natural compound called plumbagin has shown stronger anti‐proliferative effects than taxol [104]. Small peptides like NBD prevent the translocation of NFkB to the nucleus and inhibit the pathway [105]. Antisense RNA that blocks the pathway has also been tested but is still in preclinical stages of development [105]. Although efforts have been made, a lot is yet to be done in bringing effective therapeutic targets against the NFkB pathway to treat TNBC.

Several JAK/STAT inhibitors have shown potential targets in TNBC. The STAT3 inhibitor WP1066 was able to decrease proliferation and make cells chemo sensitive to doxorubicin [106]. Combined treatment of doxorubicin with tofacitinib, which is an FDA‐approved JAK inhibitor, has shown increased chemosensitivity and increased levels of apoptosis [107]. The Phase 2 clinical trials for ruxolitinib in combination with paclitaxel, doxorubicin, and cyclophosphamide are ongoing for triple‐negative inflammatory breast cancer (NCT02876302). Durvalumab, another FDA‐approved immune checkpoint inhibitor (ICI) is being tested for its efficacy to treat metastatic TNBC in combination with other drugs (NCT03742102).

**FIGURE 1:**
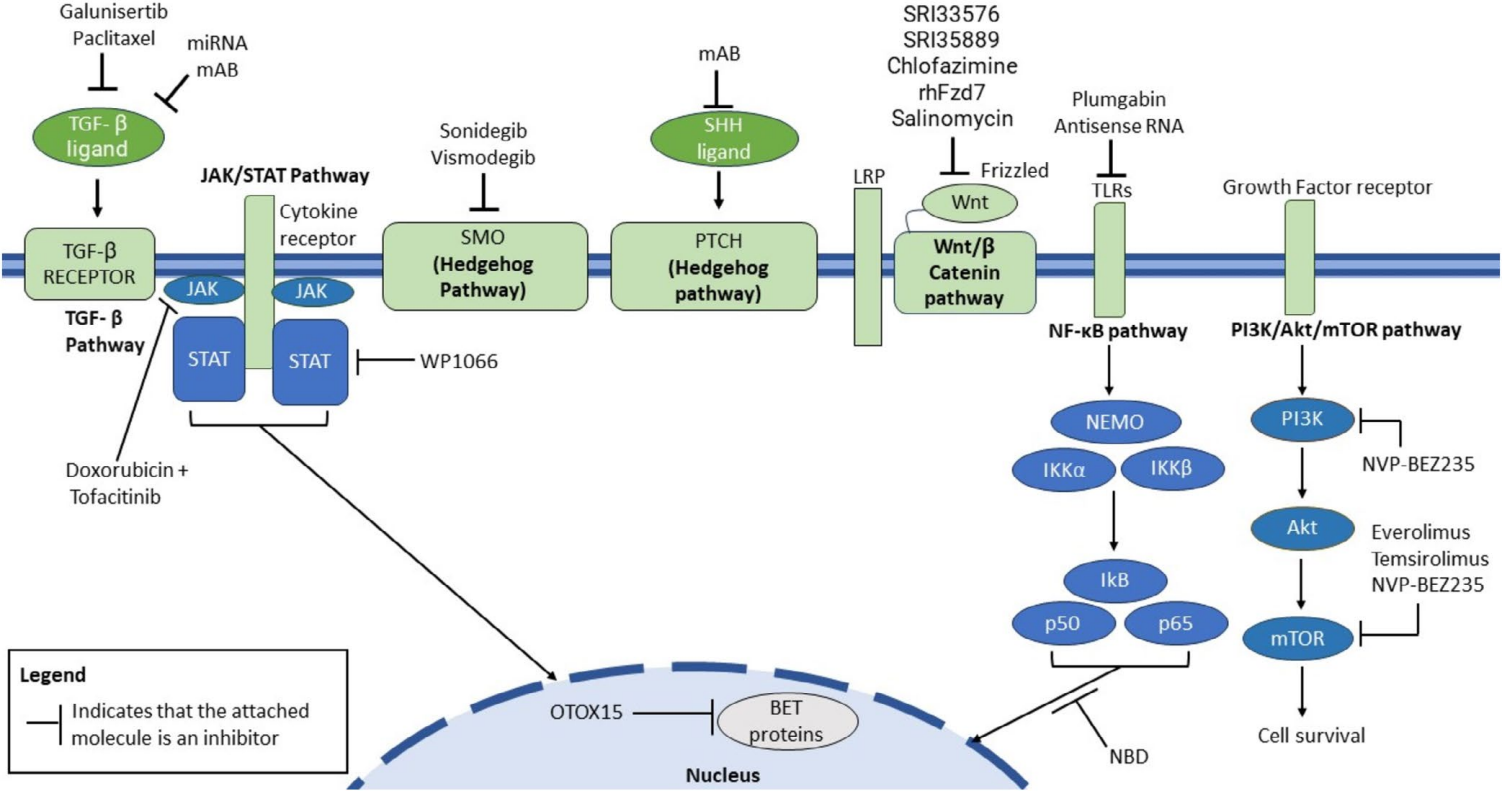
Overview of targeted cellular signaling pathways in TNBC. Various cellular signaling pathways can be targeted for the treatment of TNBC. Modes of inhibition include inhibition of receptor‐ligand binding, disruption of protein assembly, and interference with tumor signaling mechanisms, resulting in reduced cellular proliferation, reduced metastasis, and reduced tumor growth.

Drug discovery has also taken a positive course in developing new targets for the PI3/Akt/mTOR pathway. Several mTOR inhibitors, like everolimus and temsirolimus, have been evaluated and approved for breast cancers [108]. In a recent study, the combination of cisplatin with the mTOR and PI3K inhibitor NVP‐BEZ235 reversed resistance to cisplatin, reducing cell proliferation and suggesting their potential for targeted therapy [109]. The FDA has recently approved the usage of alpelisib to treat HER2 negative and advanced breast cancer [110]. The BET protein inhibitor OTXO15, used to suppress the function of oncogenes like MYC, has shown positive results, causing anti‐proliferative effects in TNBC [111]. An overview of these therapies and their mode of action is provided in Figure 1.

### 5.2 PARP Inhibitors as Therapeutic Targets

PARP inhibitors have shown promising results for several malignancies like breast and ovarian cancers. However, they might face some resistance in TNBC due to the restoration of the homologous recombination (HR) pathway and other factors that restore DNA repair functions. In a Phase 2 clinical trial of olaparib (a lethal PARP inhibitor) as monotherapy against TNBC and ovarian cancer, no significant improvement was observed in the case of TNBC [112]. However, PARP inhibitors, when combined with other forms of therapies like epigenetic modification, immunotherapy, and chemotherapy, have shown to perform better.

PARP inhibitors can work with epigenetic drugs in many different ways supported by different studies. For example, the use of HDACi along with PARP inhibitors led to a decrease in DNA damage repair and reduced proliferation in the TNBC cell lines [113]. The HDACi works by suppressing the genes involved in DNA damage response and HR pathway, enhancing the capacity of PARP inhibitors to create more DNA lesions. Another approach described by Muvarak et al. elucidated the use of a DNMT inhibitor with talazoparib, which bound tightly to the DNA, inducing double‐stranded breaks formation and cell death [114]. Apart from that, epigenetic drugs like cytidine also work by targeting the CSC population and downregulating genes like SNAIL, TWIST, and SLUG that regulate the EMT pathways [115]. Finding the right balance between combination therapies and reducing toxicity might lead to PARP inhibitors being an effective form of therapy in the case of TNBC.

### 5.3 Role of Immunotherapy in TNBC

In recent years, immunotherapy has gained popular recognition. The primary challenge encountered when implementing immunotherapy in TNBC treatment is the highly immunosuppressive nature of the TME. The TME in TNBC is found to have higher expression levels of multiple immune cell types, including CD4+ T cells, CD8+ T cells, NK cells, dendritic cells (APCs), macrophages, neutrophils, and MHC Class I proteins [116]. The presence of these cells in different subtypes of TNBC is correlated with patient prognosis and survival. Patients with higher levels of T‐lymphocytes in the tumor tissue have elevated levels of all the aforementioned cells and have better OS rates, whereas low T‐lymphocyte infiltration can be accompanied by high levels of macrophages, thus establishing that an immunosuppressive TME has to be targeted using immunotherapeutic drugs to ensure effective treatment [117]. Higher levels of certain immunoregulatory receptors also contribute to forming an immunosuppressive TME. This includes higher expression levels of CTLA4, PD‐1, PD‐L1, and others [116]. The net result of these features induces a state of hypoxia, acidosis, and oxidative stress in the TME, ultimately leading to the development of chemoresistance, metastasis, and immunosuppression.

#### 5.3.1 Role of PD‐L1 and CTLA‐4 in ICI‐Based Immunotherapy for TNBC

Immunotherapy mainly includes ICIs in the case of TNBC and is primarily geared toward suppressing CTLA‐4 and PD‐L1 expression. PD‐L1 ligands, which are found on the tumor cells, are highly expressed in TNBC [118] and interact with the PD‐1 receptors present on T cells to prevent their effector functions. About 20% of TNBC cells express upregulated PD‐L1 expression [119]. CTLA‐4 is a negative immune checkpoint that suppresses inflammatory responses, as evidenced by the higher levels of IL‐2 expression when CTLA‐4 is downregulated [120]. Pembrolizumab, atelizolumab, and nivolumab are commonly used anti‐PD1 monoclonal antibodies [121, 122]; ipilimumab and tremelimumab are anti‐CTLA‐4 antibodies being tested as mono‐therapeutic agents [123]. The efficacy of ICIs significantly improves when used in conjunction with other modes of treatment, such as chemotherapy, radiotherapy, anti‐angiogenic drugs, targeted therapy, chemotherapy, RNA vaccines, and PI3K/AKT pathway inhibitors, among others [124]. In combination with other drugs like paclitaxel and carboplatin in several clinical trials (NCT04331067), (NCT03121352), ICIs have shown to improve pCR and progression‐free survival (PFS) times.

Higher levels of PD‐L1 expression are inversely correlated with the risk of metastasis in TNBC, although joint PD‐1 and PD‐L1 interactions induce immunosuppression. Toll‐like receptors (TLRs) in TNBC cells have been found to inhibit the secretion of type I interferons, enabling the upregulation of PD‐L1. However, an increase in the basal PD‐L1 expression alone can help in recruiting TILs. The implementation of anti‐PD‐1‐PD‐L‐1 agents with neoadjuvant components, such as poly(I:C) significantly reduced the risk of metastasis and tumor burden in 4T1 and 4T2‐infected mouse models [125].

Nucleophosmin (NPM1) is a promoter for the PD‐L1 gene in cells. NPM1 is a nucleolar protein linked to key tumor‐promoting and tumor‐suppressing properties in various cancers [126]. NPM‐1 directly regulates the transcription of PD‐L1 through interactions mediated by its seven acetylation sites, with the state of acetylation causing no significant alterations to the outcome, that is, transcription and regulation of PD‐L1 [127]. Since elevated levels of PD‐L1 alone on cells initiate the recruitment of lymphocytes, we can infer that lower levels of PD‐L1 alone (unbound to PD‐1) are observed in TNBC cell lines, thus indicating modifications in the activity of NPM‐1. This hypothesis was validated by the reduced expression of PD‐L1 in mouse models infected with 4T1 cancer cells. Treatment with other agents such as olaparib inhibits the expression of PARP‐1, which enhances TIL recruitment to the TME [128].

The dysregulated expression of histone deacetylases (HDACs) can induce hypomethylation in gene transcripts, which can induce gene overexpression or silencing, thus making this an important epigenetic marker for cancer treatment, with such events being associated with the initiation of multiple types of human cancer [129]. HDAC1 is present in elevated levels in breast cancer. HDAC inhibitors (HDACi), such as vorinostat, upregulate the expression of PD‐L1 on cell surfaces, thereby enhancing TIL recruitment and counteracting immune suppression [130]. When HDACi drugs are used in conjunction with CTLA‐4 inhibitors, a significant decrease in tumor size is observed in mouse models infected with 4T1 cell lines [55].

Patients with PD‐L1 negative subtypes do not respond well to immunotherapy‐based treatments, especially those involving the usage of ICIs. However, the combination of chemotherapy or radiotherapy followed by treatment with camrelizumab, apatinib, and eribulin showed an improved overall response rate (ORR) of 37.0% in a cohort of 46 TNBC patients in a Phase II clinical trial [131]. Camrelizumab is an anti‐PD‐1 checkpoint inhibitor, and Apatinib is an anti‐angiogenic treatment that downregulates the expression of vascular endothelial growth factor 2 (VEGFR2) tyrosine kinase inhibitor. Eribulin is a chemotherapeutic drug. DC101 is a monoclonal anti‐VEGFR2 antibody. In a study that implemented a combined regimen of DC101 and anti‐PD‐1 agents, it was found that low‐level doses of DC101 sensitized tumors to TIL infiltration and treatment, that is, the tumor was less likely to develop any drug‐resistant populations and the risk of lung metastases, which is common to TNBC, was also reduced [132].

#### 5.3.2 Role of Cathepsin‐D in Immunotherapy

Cathepsin‐D is a lysosomal protease with aspartic characteristics. It is the most abundant protease in the body and plays an important role in the degradation of misfolded and aggregated proteins [133]. The elevated presence of cathepsin‐D has been associated with an increased possibility of recurrence, metastasis, and a decreased possibility of OS in breast cancer [134]. Cathepsin‐D is overproduced in TNBC patients, with higher levels of the enzyme being observed in extracellular regions of the breast tissue in TNBC patients, thus identifying it as a biomarker and therapeutic target for treatment [135].

Anti‐cathepsin‐D antibodies implemented in mouse models showed improved survival rates (49%–58%) and longer median survival times, depending on the choice of antibodies [135]. The F1 is the anti‐cathepsin‐D antibody with the highest affinity toward human TNBC cells, and the introduction of mutations in the glycosylation regions (Fc region) improves its efficacy, reduces tumor growth, and increases NK cell activity, which reduces the antibody‐dependent cellular cytotoxicity (ADCC). F1 and its engineered isoforms are unaffected by acidosis [136].

### 5.4 Oncolytic Virotherapy

Oncolytic virotherapy (OV), a novel class of immunotherapy, is gaining widespread recognition in the treatment of cancer. Viruses are modified and used to create a strong immune response against the cancer cells. They are not only helpful in recruiting lymphocytes but also promote other anti‐tumor activities like the recruitment of tumor infiltrating lymphocytes (TILs), direct lysis of tumor cells, prevention of vascularization, and also stronger immune responses by the immune system [137]. When the cells are lysed, they release damage associated molecular patterns (DAMPs) which help in the recruitment of other immune cells to tackle the tumors. The viruses can also be modified for various therapeutic advantages [137].

**FIGURE 2:**
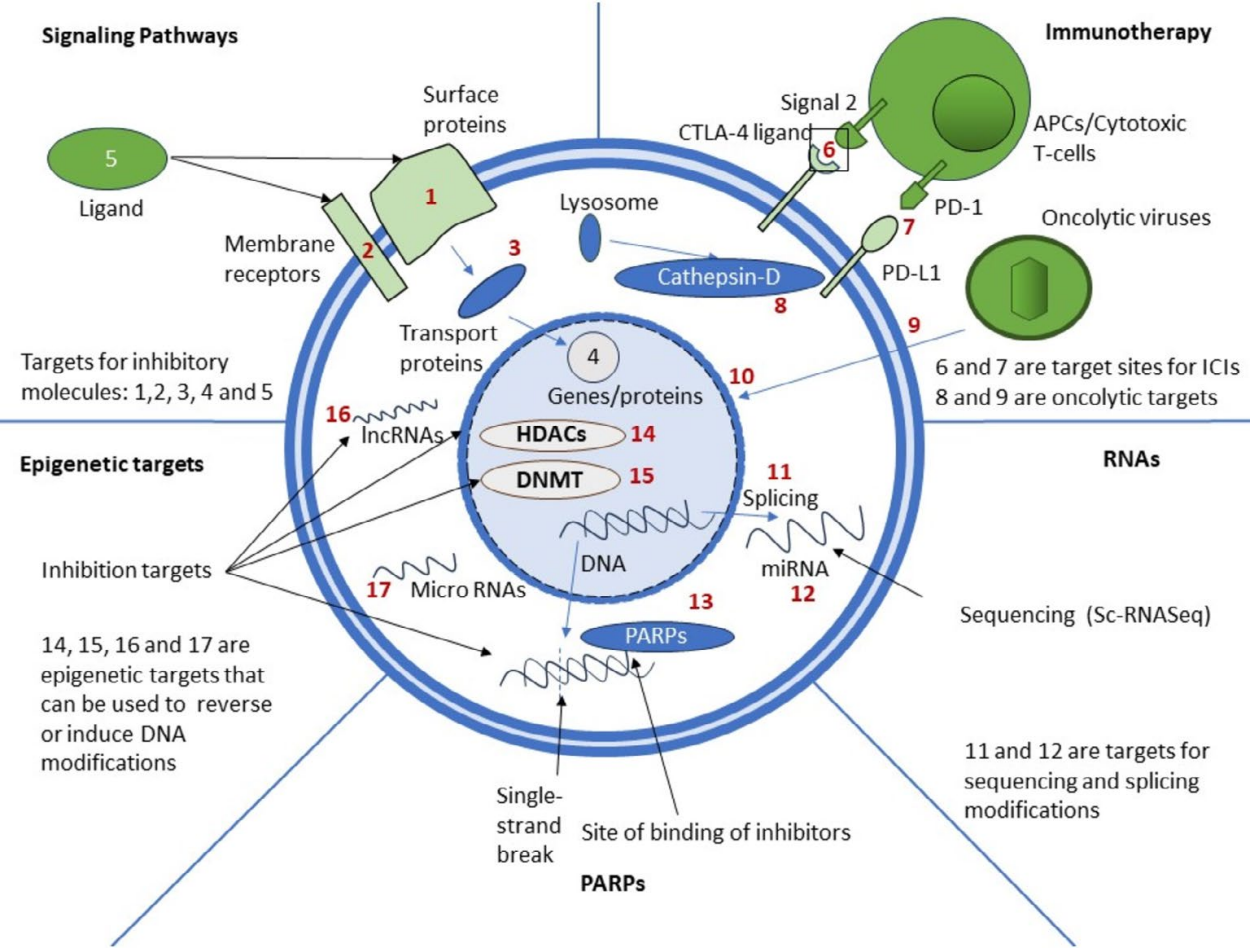
Overview of various cellular targets and therapies for TNBC. Cellular targets for TNBC treatment include signaling pathways, immune checkpoint molecules/receptors, RNAs, sites of epigenetic modifications, and PARPs. Small molecules, monoclonal antibodies, oncolytic viruses, and chemotherapy drugs are among the several treatment systems that can be used to target different components and processes in tumor cells.

In case of TNBC, several studies have been made to impact and enhance treatment efficacy. As ICIs have shown limited efficacy in case of TNBC, combining them with OV has shown better sensitivity to ICIs in case of neoadjuvant therapy [138]. Encouraging results were also observed in case of the delivery of combined therapy of a low dosage of cyclophosphamide and oncolytic adenovirus [139]. The efficacy of OV has also been tested with chemotherapy as in case of paclitaxel with Rhabdovirus Maraba MG1 in breast cancer [140]. The oncolytic herpes simplex virus has shown brilliant responses against TNBC by killing the cancer cells and raising the antitumor response due to the release of cytokine IL12 inhibiting growth and metastasis [141]. OVs have also shown effective results in reducing angiogenesis due to the production of anti‐VEGF antibody in TNBC cell lines due to the delivery of therapeutically modified vaccinia virus [142]. Temozolomide in combination with oncolytic adenovirus also shows good responses by inducing increased autophagy in TNBC cells in addition to increased viral replication and lysis of tumor cells [143]. The low toxic level and modest efficacy of OV make it a relevant candidate to fill the research gap in TNBC therapy. These cellular targets vary widely in their method of action and use, and their characteristics are summarized in Figure 2.

### 5.5 RNA Expression and Analysis in TNBC Therapeutics

#### 5.5.1 Role and Importance of miRNA as TNBC Biomarkers

miRNAs and several noncoding RNAs have proven to be particularly useful biomarkers in TNBC phenotypes that are devoid of other receptors. There are several miRNAs that are either upregulated or downregulated and provide useful evidence in identifying TNBC tumors and CSCs. Some of the upregulated miRNAs that contribute to EMT, metastasis, tumor growth, invasion, and in general a more aggressive phenotype of TNBC are miR‐10b, miR‐21, miR‐29, miR‐221/222, and miR‐373 [144]. miRNAs like miR‐205, miR‐221/222, miR‐145, and miR‐1976 repress EMT, cell migration, tumor growth, and enhance p53 activity in TNBC [144, 145]. The miR‐200 family, which is responsible for the suppression of EMT and migration [146] is often found to be downregulated in the case of TNBC [147] and is an important target for epigenetic modification using HDAC inhibitors [148]. Further, a number of miRNA signatures can perform as predictive models and as biomarkers for prognostic purposes in TNBC [149]. For example, miR‐138 is an important biomarker that is specifically expressed in TNBC and is known to promote tumorigenesis [150]. The downregulation of miR‐155 is indicative of poor prognosis and OS of patients in TNBC. Several deregulated miRNAs like miR‐130a‐3p, miR‐451, and miR‐620 also cause chemo resistance to drugs like doxorubicin and gemcitabine [151, 152].

Due to their wide‐ranging effects in TNBC, miRNAs have emerged as important therapeutic targets. There are several molecules that inhibit the oncogenic effects of miRNAs involved in TNBC. The miRNA inhibitors could be anti‐microRNA oligonucleotides (AMOs), small molecule inhibitors of miRNAs (SMIRs), locked nucleic acids (LNA), miRNA sponges, miRNA zippers, etc. [153]. The miRNA inhibitors bind to the miRNA, rendering it disabled for translation or preventing it from targeting the expression of certain genes. The co‐delivery of anti‐miR‐10b and anti‐miR‐21 using nanoparticles inhibited metastasis and anti‐apoptotic effects in TNBC mouse models [154]. miRNA molecules or miRNA mimics produced by expression vectors can be used to restore tumor suppressive functions of miRNAs in TNBC [153]. For example, Hsa‐miR‐448 suppresses KDM5B expression in TNBC, which downregulates MALAT‐mediated oncogenic functions [155]. The combined therapy of miRNA mimics with other anti‐cancer treatments can also result in enhanced sensitivity to chemo and radiotherapy [156]. However, although miRNAs are extremely essential targets for treating TNBC, several different aspects of toxicity, delivery mechanisms, and specificity still remain to be explored.

Apart from these, several lncRNAs like ANRIL, UCA1, HOTAIR, and MALAT1 also have oncogenic potential, enhancing metastasis, EMT, CSC proliferation, and poor prognosis in patients [157]. They also serve as biomarkers for diagnosis and targeting, which can antagonize several tumor‐associated mechanisms [158]. For example, MNX1‐AS1 is an upregulated lncRNA which, when silenced, controls the aggressiveness in TNBC [159]. Another example is KDM5B, which epigenetically modifies lncRNA MALAT1 expression, leading to tumor formation, invasion, and CSC phenotypes in TNBC [160]. lncRNAs also encourage chemoresistance, enhance drug efflux, and promote the escape of cells from apoptosis. They can be targeted by small molecule inhibitors using anti‐sense oligonucleotides, siRNAs, hammerhead ribozymes, and crispr‐cas9 to enhance therapeutic efficacy. Research on lncRNAs is still in its nascent stages and needs further exploration to fully utilize and understand their implications [161]. The role and behaviour of various microRNAs in TNBC has been summarized in Table 2.

**TABLE 2:** Role of various micro RNAs in TNBC.

Micro RNA	Pattern of expression	Role in TNBC	References
miR‐10b	Differential levels of expression in TNBC and ER(+)/PR(+) were not observed	Has been linked to metastasis, cell invasion and migration in advanced tumors Links to normal development and prognosis remain under scrutiny.	[162, 163, 164]
miR‐21	Differential levels of expression in TNBC and ER(+)/PR(+) were not observed. However, miR‐21 is overexpressed in TNBC as opposed to normal breast tissue	High levels of miR‐21 have been correlated with decreased chances of PFS It also promotes cell invasion and metastasis, and interferes negatively with tumor inhibiting processes, such as apoptosis in the body	[165, 166, 167, 168]
miR‐29	miR‐29a is upregulated in breast cancer tissue Levels of miR‐29b are highly elevated in all metastatic forms of breast cancer, with the level of elevation being positively correlated to the extent of metastasis^a^ miR‐29c levels decrease as the cancer advances	miR‐29a plays a role in promoting EMT, cell migration, and metastasis miR‐29b plays a role in regulating treatment response, angiogenesis, and metastasis miR‐29c inhibits colony formation, cell proliferation, and metastasis	[169, 170, 171]
miR‐221/222	miR‐221/222 are upregulated in TNBC	Higher levels are associated with a poor prognosis, promote oncogenesis, EMT and can reduce cell sensitivity to chemotherapy	[172, 173]
miR‐373	Upregulated in TNBC tumors and benign masses, levels in the former are higher	Inhibits CD44 to promote cell invasion metastasis, and cell migration in metastatic cancer subtypes. Is also a prognostic factor	[174, 175, 176]
miR‐205	Downregulated in TNBC, and levels of miR‐205 are inversely correlated with the metastatic nature of the tumor	Regulates cell proliferation, EMT, metastasis. Biomarker for determining ER/PR state of the tumor. Also suppresses CSC formation and proliferation. Overall survival rates improve as levels of miR‐205 increase	[177, 178, 179]
miR‐145	Downregulated in breast cancer, although levels in ordinary carcinoma cells are lower than those of TNBC	miR‐145 inhibits cell migration, invasion, motility and metastasis. It promotes apoptosis through cytokine upregulation	[144, 180, 181]
miR‐1976	Downregulated in TNBC	Inhibits cell invasion, migration and metastasis, can be used as a prognostic factor for predicting survival	[145]
miR‐200	Downregulated in breast cancer and TNBC	Inhibit factors that promote angiogenesis, proliferation, migration, invasion and metastasis, and suppresses EMT/MET. It is directly correlated with OS and prognosis^b^	[182, 183, 184]
miR‐138	Upregulated in TNBC, downregulated in malignant breast cancer (metastatic and non‐metastatic)	Promotes cell viability through increased proliferation and inhibiting apoptosis	[150, 185]
miR‐155	Upregulated in TNBC	Higher levels are believed to induce chemoresistance, and confers stem‐cell characteristics to tumor cells, thereby promoting survival and proliferation	[186, 187]
miR‐130a‐3p	Downregulated in TNBC and breast cancer	Inhibits cell proliferation (arrests cell growth) and growth in the absence of solid media (anchorage independent growth) and cell migration if overexpressed. Overexpression can improve sensitivity to chemotherapeutic agents by increasing rates of apoptosis	[152, 188, 189]
miR‐451	Downregulated in TNBC and breast cancer	Can improve chemosensitivity by reducing cell proliferation, migration, invasion, and promoting apoptosis. Decreased levels indicate lower OS	[152, 190, 191]
miR‐620	Upregulated in breast cancer tissues	Promotes tumor cell progression, and confers resistance to chemotherapy	[151, 192]

Abbreviations: CSC, cancer stem cell; EMT, epithelial–mesenchymal transition; ER, estrogen receptor; MET, mesenchymal–epithelial transition; miR, micro RNA; OS, overall survival; PFS, progression‐free survival; PR, progesterone receptor; TNBC, triple‐negative breast cancer.

^a^ While this general conclusion can be derived, it has to be noted that different forms of miR‐29b may be upregulated or downregulated in TNBC but are correlated with the same effects nonetheless.

^b^ These functions are defined with respect to miR‐200c, although miR‐200s have been postulated to be highly accurate biomarkers in their entirety.

#### 5.5.2 Single Cell RNA Sequencing and Epigenetic Analysis in TNBC

Unraveling effective targeted therapy in the case of TNBC is majorly dependent on understanding the heterogeneity in the type of cells being exposed to different microenvironments in a population. Techniques like single‐cell RNA sequencing (ScRNA‐Seq) and epigenetic analysis of the tumors in TNBC will help us understand the intricacies of each subtype. In a stem cell population, each type of cell varies in its expression of gene patterns, which escape notice when we dissect and study bulk tissues [193]. The epigenetic regulation of cells also plays an important role in generating stem cell‐like properties, all of which need to be accounted for while looking for targets in TNBC.

ScRNA‐Seq has led to the identification of different classes of stem cells with unique isoform splicing patterns. Even in the case of TNBC, heterogeneous expression of the supposedly absent receptors (ER, PR, and HER2) has been observed across different cells. The three different classes of stem cell populations observed were EMT CSCs, MET (mesenchymal‐epithelial transition) CSCs, and dual EMT‐MET CSCs. Apart from these, EMT non‐CSCs and non‐CSCs were also observed. The variations in the splicing patterns not only contribute to making the tumors more diverse and difficult to study but also enhance heterogeneity. For example, in some cells, heterogeneity is observed in terms of the expression of TNBC marker genes ERBB2, ESR2, etc. ranging from complete absence of the two to no simultaneous expression of both in the cells. The higher expression of the ESR isoform ESR‐208 promotes the degradation of Erα, and its inhibition might lead to the conversion of TNBC to ER + ve, opening up targeted therapy options [194]. Some cells also express HER2 receptors, which confirms the conversion of stem cells to HER2‐positive type and helps us extend HER2‐targeted treatment options to a specific subset of cells in TNBC [195]. Another important observation is that the isoform CD44v6 is particularly linked with proliferation and tumor progression in TNBC. It has also been found that TNBC tumors share a subpopulation of cells that have the same biological features in all patients. Also, a particular malignant subpopulation of cells has been identified that is a driver for low pCR and aggressive phenotypes. A particular glycosphingolipid pathway also plays a role in predicting patient outcomes. S1PR1, a gene involved in this pathway, has been implicated as an essential therapeutic target [196]. A deeper understanding of the extreme heterogeneity of CSCs in TNBC will lead to the development of novel therapeutics directly targeting the different subpopulations of CSCs in breast cancer. The therapies discussed so far are rather complex in their fabrication and implementation and are at various stages of clinical trials or laboratory testing and research (see Table 3).

**TABLE 3:** List of drugs and molecules currently being tested for TNBC treatment.

Drug	Type	Target gene/receptor	References/clinical trial identifier
Chemotherapy drugs			
Paclitaxel	Chemotherapy drug (Taxane)	Apoptosis regulators, tubulins, microtubule‐associated proteins	[25]
Gemcitabine	Chemotherapy drug	DNA, UMP‐CMP kinase, thymidylate synthase, ribonucleoside‐diphosphate reductase large subunit	[25]
Docetaxel	Chemotherapy drug	Microtubules	[34]
Doxorubicin	Chemotherapy drug	Topoisomerase II	[44, 45]
Azacytidine	Epigenetic drug/chemotherapy drug	SNAIL, TWIST, and SLUG	[114]
Eribulin	Chemotherapy	Microtubules	[55]
Temozolomide	Chemotherapy	DNA	[108, 143]
SMO inhibitors			
Galunisertib^a^	Small molecule inhibitor (TGF‐βR1 kinase inhibitor)	Growth factors, e.g., TGF‐β	NCT02672475
Vismodegib	SMO inhibitor (cyclopamine‐competitive antagonist of HH pathway)	SMO	NCT02694224
Sonidegib	SMO inhibitor	SMO	[34]
Wnt/β‐catenin pathway inhibitors			
SRI33576	Wnt/β‐catenin pathway inhibitor	LRP6, cyclin D1 and active β‐catenin	[35]
SRI35889	Wnt/β‐catenin pathway inhibitor	LRP6, cyclin D1 and active β‐catenin	[36]
Salinomycin	Wnt/β‐catenin pathway inhibitor, ABC drug transport inhibitor	ABC transporter	[39]
rhFzd7	Wnt/β‐catenin pathway inhibitor	Wnt3a	[41]
JAK/STAT pathway inhibitors			
WP1066	STAT3 inhibitor	STAT3	[42, 43]
WP1066	STAT3 inhibitor	STAT3	[42, 43]
Tofacitinib	JAK inhibitor	JAK	[43, 44]
Ruxolitinib	JAK inhibitor	JAK	NCT02876302 [43]
mTOR/Akt/P13K pathway inhibitors			
Everolimus	mTOR inhibitor	mTOR	[46]
Temsirolimus	mTOR inhibitor	mTOR	[46]
NVP‐BEZ235 (Dactolisib)	mTOR and P13K inhibitor	mTOR and P13K	[46, 47]
Alpelisib	α‐subunit‐specific PI3K inhibitor	Class I PI3K p110α	[46, 48]
Drugs targeting other cellular pathways			
NBD (NEMO‐binding domain)	NF‐kB pathway inhibitor	NEMO proteins	[43, 103]
Tarextumab	Anti‐notch signaling	Notch 2/3 receptors	[36, 44, 45]
Immune checkpoint inhibitors			
Pembrolizumab	ICI	PD‐1	[113, 120]
Nivolumab	ICI	PD‐1	[114, 120]
Ipilimumab	ICI	CTLA‐4	[120, 121]
Tremelimumab	ICI	CTLA‐4	[120, 121]
Camerlizumab	ICI	PD‐1	[55, 121]
Durvalumab	ICI	PD‐L1	NCT03742102 [44]
Tumorigenic process inhibitors			
Apatinib	Anti‐angiogenic treatment	VEGFR‐2 tyrosine kinase	[55, 129]
DC101	Anti‐angiogenic treatment	VEGFR2	[55, 132]
Myricetin	Anti‐oxidant agent	Signaling networks	[156, 157]
Oncolytic virotherapy drugs			
Maraba MG1	Oncolytic virotherapy	Dendritic cells	[132, 140]
Herpes simplex virus	Oncolytic virotherapy	Cancerous cells	[136, 141]
HDAC inhibitors			
MS‐275	HDAC inhibitor	HDAC	[48, 143, 197]
Vorinostat	HDAC inhibitor	PD‐L1 expression/genes	[77, 129]
SGI	DNMT inhibitor	DNMT	[48, 141, 198]
P13/Akt pathway‐targeting drugs			
Fisetin	P13/Akt pathway target	P13/Akt pathway	[48, 156, 198]
Quercetin	P13/Akt pathway target	P13/Akt pathway	[48, 156, 197]
Survivin inhibitors			
Selinexor	Survivin inhibitor	XPO‐1	[156, 199]
miR‐34a	Survivin inhibitor	Survivin	[157, 199]
Other protein inhibitors			
DAPT	γ secretase inhibitor	γ secretase	[34, 35]
OTOX15 (Birabresib)	BET protein inhibitor	BD1 and BD2 (bromodomains), c‐MYC	[47, 49]
BIO	GSK3β inhibitor	GSK3β	[48, 55]
Hsa‐miR‐448	KDM5B inhibitor	KD5MB	[49, 105]
Talazoparib (Talzenna)	PARP inhibitor	PARP 1 and PARP 2	[55, 113]
F1	Anti‐cathepsin‐D treatment	Cathepsin‐D	[55, 136]
Dyes and toxins			
Chlofazimine	Riminiphenazine antimycobacterial dye	Wnt3a	[39, 103]
Plumbagin	Plant toxin	Caspase‐3	[41, 42]

Abbreviations: ABC, ATP‐binding cassette; Akt, protein kinase B; BD, bromodomain; BET, bromodomain and extra‐terminal domain; CMP, cytidine monophosphate; CTLA4, cytotoxic T‐lymphocyte‐associated protein 4; DAPT, N‐[N‐(3,5‐difluorophenacetyl)‐L‐alanyl]‐S‐phenylglycine t‐butyl ester; DNA, deoxyribonucleic acid; DNMT, DNA methyltransferase; GSK, glycogen synthase kinase; HDAC, histone deacetylases; HH, hedgehog; ICI, immune checkpoint inhibitor; JAK, Janus Kinase; KDM5B, lysine demethylase 5B; LRP, low‐density lipoprotein receptor‐related protein; miR‐34a, micro RNA‐34a; mTOR, mammalian target of rapamycin; NBD, NEMO‐binding domain; NEMO, NF‐κ‐B essential modulator; NF‐κ‐B, nuclear factor kappa B; P13K, phosphoinositide 3‐kinase; PARP, poly(ADP‐ribose) polymerases; PD‐L1, programmed death ligand 1; SMO, smoothened; STAT, signal transducer and activator of transcription; TGF, transforming growth factor; UMP, uridine monophosphate; VEGFR, vascular endothelial growth factor receptor; Wnt, wingless‐related integration site; XPO1, exportin 1.

^a^ Discontinued in 2020 by Eli Lilly, the manufacturer, due to concerns regarding disease progression [200].

### 5.6 Emerging Potential of Phytochemicals and Natural Compounds

**FIGURE 3:**
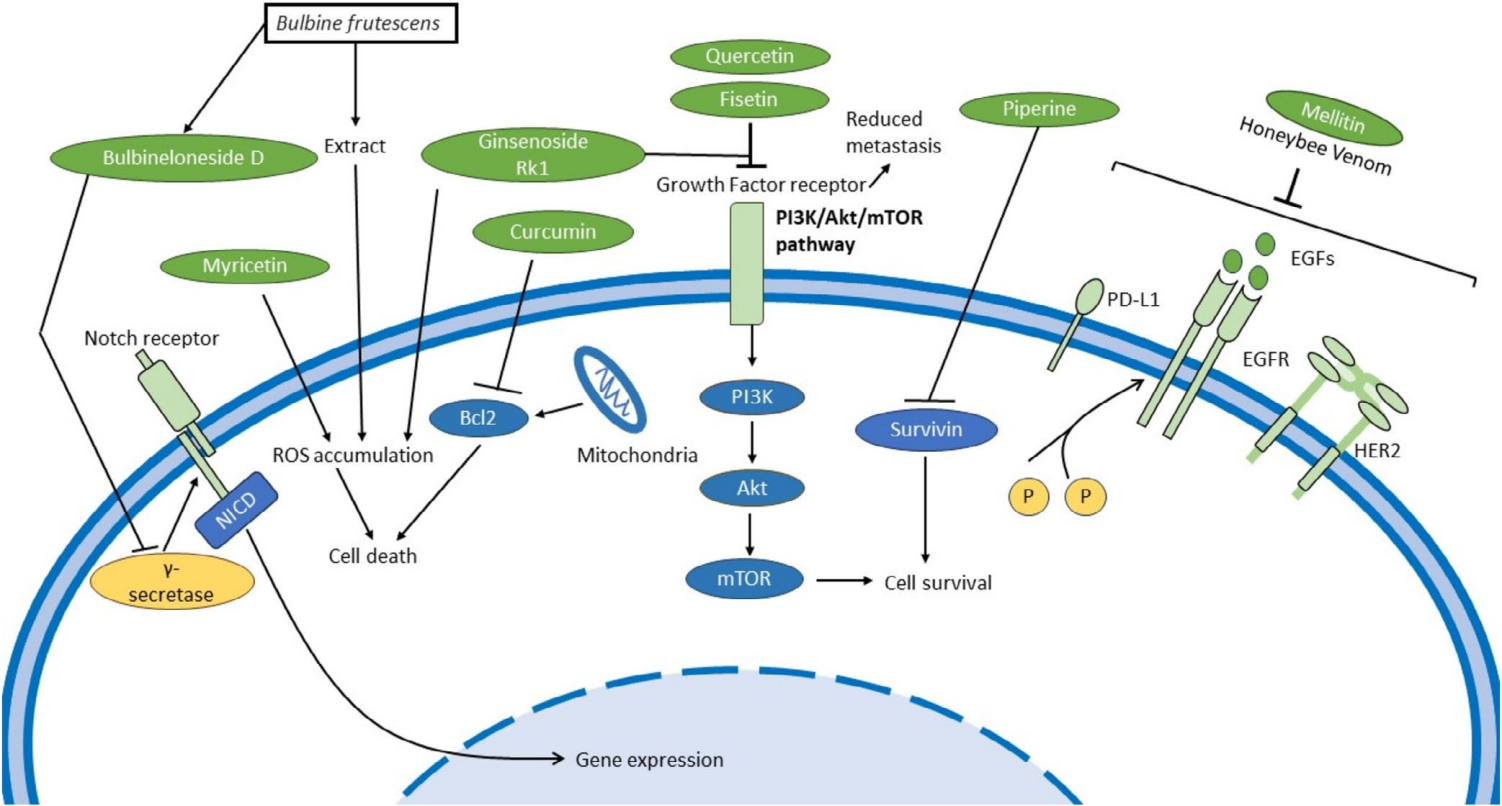
Phytochemicals used for TNBC treatment and their cellular targets. Phytochemicals have been shown to play an important role in future treatments for TNBC. Plant compounds and their derivatives may be used alone or in conjunction with standard treatments, such as chemotherapeutic drugs, to treat TNBC. Phytochemicals are capable of reducing metastasis, inducing apoptosis by triggering the required cascades and inducing ROS accumulation, and inhibiting the activation of cellular signaling pathways.

The role of phytochemicals in either eliminating or complementing standard therapy cannot be ignored in a form of cancer with such an aggressive phenotype. Phytochemicals have shown encouraging results in decreasing tumor burden and metastasis and have different cellular targets, as elucidated in Figure 3. Fisetin and Quercetin are two phytochemicals that have shown reduced metastasis in the case of TNBC, mostly by targeting the components of the PI3/Akt pathway [201]. Myricetin, another phytochemical, leads to the accumulation of ROS inside the cell, leading to cell death in TNBC [202]. Ginsenoside RK1, an anticancer compound, was also found to reduce tumor growth, enhancing apoptosis by targeting similar pathways [199]. *Bulbine frutescens* is another plant whose compounds promote cell cycle arrest by downregulating the notch pathway [203]. The exceptional role of curcumin and its derivatives in inducing apoptosis and effectively killing tumor cells has also been implicated in reducing the size of TNBC tumors [204]. In a very recent trial, the effect of apitherapy was brought to light, where the effect of Melittin and honeybee venom was explored to treat aggressive cancer cells like those of TNBC. They inhibit the phosphorylation of EGFR and HER2 receptors and reduce cell viability at low toxicity. Further, they have not only supported the enhanced efficacy of chemotherapeutic drugs like docetaxel, but by reducing the expression of PDL1 ligands, they can also be beneficial in the effects of immunotherapy [205]. Phytochemicals are also useful when targeting proteins such as survivin, which have been implicated in cell proliferation and metastasis in TNBC. Piperine is an emerging phytochemical believed to have a significant effect on CSCs, as seen in the case of glioblastoma, where low doses of piperine in combination with temozolomide (TMZ) were found to reduce cellular migration, stemness, and survivin expression, thus highlighting a potentially novel treatment [206]. However, significant alterations in cell apoptosis levels were not observed during the treatment period in vitro. These findings are applicable to TNBC as well, as elucidated by a 2015 study conducted by Greenshields et al. [207]. This study showed that higher concentrations of piperine could be used to reduce cell migration and induce apoptosis in xenograft models and were found to improve the sensitivity of TNBC cells to radiation treatment. These findings support their efficacy and lead us to invest in and investigate more into the role of anticancer phytochemicals as cheaper and reliable treatment options. Further investigation can serve to overcome several significant challenges encountered with the use of phytochemicals as a standalone or adjuvant treatment in breast cancer. These challenges include extraction, where seasonal and regional factors, such as soil condition, water content, and methods used for extraction and harvesting, can significantly alter the kinetics and quality of the product obtained [208]. Additional issues constitute the applicability of phytochemicals in clinical studies, where the difficulty in the standardization of product quality, dose, and extraction, in addition to heterogeneous responses as a result of large disparities in results obtained from in vivo and in vitro or ex vivo studies, deters their widespread implementation as a standard treatment modality [209, 210].

## 6 Conclusion

The average 5‐year survival rate of TNBC is 77%, as per the American Cancer Society. The type of tumor and its progression determine the viable options for treatment. Tumor resection alone is insufficient as a treatment option, as bone and lung metastases are common with advanced stages of TNBC. Biomarker identification and targeting remain major roadblocks in the treatment of TNBC. Chemoresistance is extremely prevalent, with patient response to SoC treatments declining steadily, highlighting the need to identify alternative modes of treatment. These modes of treatment include ICIs, phytochemicals, use of ABC transporters, cellular signaling pathway inhibitors, and OV, etc. They are often used in combination with conventional chemotherapy and/or radiotherapy to improve efficacy and reduce the risk of remission and tumor metastasis. Their primary advantages lie in their ability to target and inhibit baseline cellular processes that lead to the development of undesirable traits, such as chemoresistance and plasticity. Advancements in these areas have been promising, indicating the possibility of improved options for treating patients diagnosed with TNBC. Ongoing clinical trials experiment with various combinations for treatment, and epigenetic databases help streamline the determination of an appropriate, personalized course of treatment. These trials look at implementing different treatment courses and test the efficacy of combined chemotherapy and novel therapeutics or novel therapeutics alone, with developments favoring combined therapies, with concerns regarding non‐specific cellular toxicity due to chemotherapeutic drugs and resistance to treatment being alleviated. The inclusion of appropriate targets for effective therapy, such as survivin, especially in CSCs, has the potential to combat chemoresistance and metastasis. The combination of such therapeutics has the potential to improve patient survival in TNBC. Understanding the epigenetic profiling of TNBC‐affected populations opens up new avenues for treatment, including reversal of epigenetic modifications, inhibition of gene expression, etc. New platforms for delivery, including nanoplatforms or nanocarriers, which can be engineered to target various biomarkers while reducing the toxicity of existing treatments, may be explored in the future, along with personalization in treatment protocols to improve patient survival. Nanocarriers may emerge as the optimal mode of delivery for several solid tumors, as they are able to carry several therapeutics of varying importance in a very precise manner. A thorough and complete characterization of various techniques and their clinical efficacy is essential in improving the scope of the discussed treatments for TNBC, with the vast array of emerging treatments heralding an improved treatment landscape, wherein various aspects, such as personalization and precision come to the fore.

## Author Contributions

**Rachana Raman:** visualization (lead), writing – original draft (lead), writing – review and editing (lead). **Shristi Debata:** writing – original draft (supporting). **Thirupugal Govindarajan:** writing – review and editing (supporting). **Praveen Kumar:** conceptualization (lead), writing – review and editing (lead).

## Conflicts of Interest

The authors declare no conflicts of interest.

## Data Availability

The authors have nothing to report.
